# Application of improved grey wolf model in collaborative trajectory optimization of unmanned aerial vehicle swarm

**DOI:** 10.1038/s41598-024-65383-9

**Published:** 2024-07-27

**Authors:** Jiguang Chen, Yu Chen, Rong Nie, Li Liu, Jianqiang Liu, Yuxin Qin

**Affiliations:** 1 School of Electronics and Information, Zhengzhou University of Aeronautics, Zhengzhou, 450046 China; 2 Collaborative Innovation Center of Aeronautics and Astronautics Electronic Information Technology, Zhengzhou University of Aeronautics, Zhengzhou, 450046 Henan Province China; 3 Henan Key Laboratory of General Aviation Technology, Zhengzhou University of Aeronautics, Zhengzhou, 450046 China

**Keywords:** Grey wolf algorithm, Deep reinforcement learning, Unmanned aerial vehicle, Track planning, Swarm intelligence optimization, Computer science, Information technology

## Abstract

With the development of science and technology and economy, UAV is used more and more widely. However, the existing UAV trajectory planning methods have the limitations of high cost and low intelligence. In view of this, grey Wolf algorithm is being used to achieve collaborative trajectory optimization of UAV groups. However, it is found that the Grey Wolf optimization algorithm (GWO) has the problem of weak cooperation. In this study, based on the traditional GWO pheromone factor is introduced to improve it.. Aiming at the problem of unstable performance of swarm intelligence optimization algorithm under dynamic threat, deep reinforcement learning is used to optimize the model. An unmanned aerial vehicle swarm trajectory planning model was constructed based on the improved grey wolf algorithm. Through experimental analysis, the optimal fitness value of the improved grey wolf algorithm was lower than 0.43 of the grey wolf algorithm. Compared with other algorithms, the fitness value of this algorithm is significantly reduced and the stability is higher. In complex scenarios, the improved grey wolf algorithm had a trajectory length of 70.51 km and a planning time of 5.92 s, which was clearly superior to other algorithms. The path length planned by the research and design model was 58.476 km, which was significantly smaller than the other three models. The planning time was 5.33 s and the number of path extension points was 46. The indicator values of the Unmanned Aerial Vehicle swarm trajectory planning model designed by this research were all smaller than the other three models. By analyzing the results, the model can achieve low-cost trajectory optimization, providing more reasonable technical support for unmanned aerial vehicle mission execution.

## Introduction

Unmanned Aerial Vehicle (UAV) can directly replace humans to complete some special air missions. Compared to manned vehicles, UAV is actually safer in extreme environments and can avoid accidents during task execution^1^. With the development of UAV and the increasing demand for it by humans, UAV faces more challenges and requires more capabilities. The increasingly complex tasks and environments require UAV systems to have high autonomy^2^. When performing flight missions, UAV urgently needs reliable and reasonable trajectory planning algorithms to calculate feasible paths to avoid terrain obstacles and enemy threats encountered to ensure the smooth completion of flight missions^3^. The static threat during the UAV flight is fixed and only needs to be planned once. But for dynamic threats, each threat is not fixed and has uncertainty, dynamism, and variability^4^. In recent years, due to the development of electronic counter-measures and intelligent technology, it is very important to improve the anti-interference capability of UAV navigation. However, traditional trajectory planning methods cannot guarantee the accuracy and real-time performance in complex environments, and their intelligence is not high^5^. To this end, a new UAV cluster trajectory planning optimization model is constructed using Grey Wolf optimization algorithm (GWO) and Deep Reinforcement Learning (DRL). This provides a flight path for improving the UAV electronic warfare, starting from the starting point and following the optimal flight path to reach the target point.

The use of reinforcement learning for trajectory replanning is investigated in response to the dynamic adjustment of UAV swarm tasks in emergency adjustment scenarios. A modified strategy using deep deterministic strategy gradient algorithm for actual trajectory planning is proposed to achieve dynamic optimization of trajectory planning models. There are two innovative points in the research. The UAV trajectory planning environment and framework are constructed based on the threat sources in the flight space. The combination of swarm intelligence optimization algorithm and DRL achieves the UAV trajectory planning. The study consists of four parts. Firstly, there is a literature review, which summarizes the techniques and domains used in the research. Secondly, a detailed introduction is given to the techniques used in the trajectory planning optimization model and the model construction. In the third section, comparative experiments on the performance of the designed model are performed and the correspondence conclusions are drawn. Finally, the research content and performance analysis results are summarized, and the research prospects are proposed.

## Related works

The complex and constantly changing obstacle environment poses significant challenges to the navigation safety of UAV, and the trajectory planning has gradually become a key research direction for scholars. B. Li et al. proposed a UAV data distribution system with expected data requirements to address the low endurance of air-borne batteries in harsh environments. By evaluating the relationship between task time and propulsion energy consumption, a more energy-efficient navigation trajectory was designed^6^. Based on the chance constrained optimization methods, B. Du et al. studied UAV trajectory planning with probabilistic geofencing and solved the formula using a novel sampling-based solution method. An iterative scheme was introduced into the algorithm to obtain the optimal trajectory to obtain a completely collision free trajectory^7^. Y. C. Ko et al. proposed a method for designing UAV velocity functions and trajectories. Firstly, a method of actively selecting the UAV shooting altitude was adopted, a UAV function was designed, and the UAV flight time for all areas was derived. Then the ground access sequence was derived based on the flight time. Finally, the candidate coverage paths were generated^8^. E. Akin et al. developed an estimation planning algorithm based on multi-agent Q-learning to achieve effective and fast situational awareness through UAV during post-disaster reconstruction. This algorithm maintained all-weather connectivity with ground stations in a multi-hop manner and uses UAV to observe as many critical areas as possible^9^. H. Gao et al. studied two approaches of the multi-UAV trajectory planning and resource allocation to improve the freshness of the collected information. The exploration and learning ability of the dual delay deep deterministic strategy gradient algorithm were utilized for the UAV trajectory planning, and the optimal resource allocation was achieved using the round robin rule^10^.

GWO has significant advantages in terms of accuracy and convergence speed, which has a wide range of application areas. J. Du et al. pointed out that traditional Grey Wolf Optimizer (GWO) was prone to falling into local optima, resulting in low quality scheduling schemes. They proposed a scheduling model that took into account the comprehensive demand response of electricity and heat. Based on the traditional GWO, fuzzy C-means clustering algorithm was used to group wolves, increase population diversity, and obtain an improved GWO to achieve optimal scheduling of energy systems^11^. V. Rajagopal et al. proposed a control algorithm based on synchronous reference frame theory for load balancing in the grid-connected photo-voltaic systems. This algorithm utilized the gain of the GWO controller. Compared with existing optimization algorithms, this algorithm had excellent performance and a control accuracy of 0.925^12^. Q. Zhang et al. proposed a robot path planning optimization method based on the Improved Grey Wolf Algorithm (I-GWO) and self-powered sensor technology to address the low efficiency in the optimal path planning for existing mobile robots. The proposed optimization algorithm had improved the optimization effect by 14.84% compared to other algorithms through experimental analysis^13^. D. S. Khafaga et al. proposed a zoo dataset classification model based on particle swarm and GWO to improve the accuracy of data classification. How different voting procedures affected the efficiency of two different classification algorithms was studied^14^. K. N. Rao et al. proposed a novel modified GWO based on fitness update evaluation to design an optimized Vivaldi antenna to improve antenna zengy6i, using an optimization algorithm to fine tune antenna parameters. And the proposed algorithm was compared with traditional algorithms in terms of gain, standing wave ratio, directivity, charge, and current distribution^15^.

Based on the above content, GWO achieves superior performance in trajectory planning. However, most of the above literature does not fully address the problem of fixed-wing UAV clusters with high latitude and multiple constraints, and there are limitations in the applicability. Therefore, pheromone factors have been introduced into traditional GWO to address static and dynamic trajectory planning for UAV swarms in different complexity scenarios. The article aims to provide more efficient path planning solutions for UAV swarm in fields such as disaster relief and terrain exploration.

### Construction of a trajectory optimization model for unmanned aerial vehicle swarm based on i-gwo and reinforcement learning

As modern technology develops, traditional UAV trajectory planning methods are no longer able to meet the needs of their flight missions. The complex and ever-changing flight environment has also made the UAV trajectory planning very difficult. To this end, a UAV swarm collaborative trajectory planning model is constructed using I-GWO and reinforcement learning. The following is a description of the model construction and the techniques used.

### Proposal of unmanned aerial vehicle trajectory planning issues

The fixed wing UAV is considered in the study, which is known for its high speed, long range, and large load capacity. It is suitable for long-range reconnaissance, target tracking, and intelligence gathering. Dupin turn is a complex flight maneuver, which is usually used for fast turning of fixed wing UAV in limited space. It requires UAV to have high maneuverability and flight control system to achieve accurate trajectory control. The use of Durbin gyrates in fixed-wing UAV does add operational complexity, but it also allows the UAV to be more flexible in response to a variety of mission requirements. The representation method of UAV flight trajectory is often related to actual flight tasks and problem requirements^16–18^. For this, the study uses trajectory points as three-dimensional (3D) representations. Because UAV needs to further ensure the success rate of planning when facing new threat information, the final optimal trajectory is presented through a smoother curve. The specific trajectory is represented by Eq. (1).

1 $$ L = \left\{ {A,P_{1} ,P_{2} ,...,P_{n} ,B} \right\} $$

In Eq. (1), $A$ is the starting position of the flight. $P_{1}$ and $P_{2}$ are both trajectory points in the planning process. $B$ is the target location. In the UAV trajectory planning, there are four main aspects to consider: environmental information, planning methods, trajectory, and flight constraints. This can realistically reflect the actual flight situation of UAV and meet its feasibility requirements, thus making the planned trajectory realistic and reliable. The maximum range, flight altitude threshold, flight speed, turning angle, and minimum turning radius are chosen as constraints. The flight area of UAV is a relatively vast 3D space. The UAV's own motion constraints and spatial threat information are combined to set the position coordinates of UAV in the 3D flight space. Common spatial planning expression methods include discrete grid method and continuous space Voroni diagram, visual graph, continuous method, etc. The connecting line method is a planning method that optimizes the starting point segment by segment, with advantages such as simple implementation and short solving time. For ease of description, the study uses the line method for 3D spatial planning. The 3D planning space is represented by Eq. (2).

2 $$ C = \left\{ {\left( {x,y,z} \right)|0 < x < x_{\max } ,0 < y < y_{\max } ,0 < Z < Z_{\max } } \right\} $$

**Figure 1:**
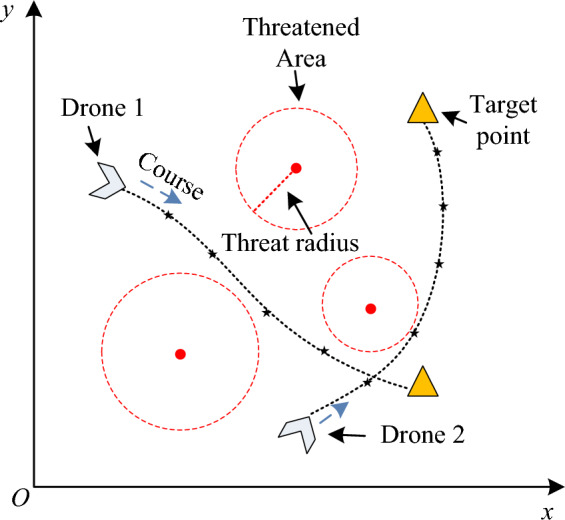
Two-dimensional navigation trace diagram of the UAV.

In Eq. (2), $x$ is the longitude of UAV in the space. $y$ is the latitude of UAV in the space. $z$ is the altitude. A schematic diagram of the UAV two-dimensional trajectory is shown in Fig. 1.

The radius of each threat area is represented by $R_{m}$. The probability of being attacked upon entering the threat area will also increase considering that multiple fixed wing UAV need to cross multiple threat sources with different threat levels and fly to the target point. The radius is used to represent the threat coverage range. The study categorizes the threats encountered in UAV missions into three types: enemy radiation sources, terrain obstacle threats, and flight environment weather. The study adopts numerical quantification to represent threats. For the same threat, the corresponding threat probability will change with the change of the radius of action. The study multiplies the height corresponding to the radius of action by the threat probability to obtain the threat cost. The corresponding data of height are represented by Eq. (3).

3 $$ T\left( {x,y} \right) = \sum\limits_{i = 1}^{M} {T_{i} \left( {R_{m}^{2} - \frac{{\left( {x - x_{0i} } \right)^{2} }}{{x_{si} }} - \frac{{\left( {y - y_{0i} } \right)^{2} }}{{y_{si} }}} \right)} $$

In Eq. (3), $\left( {x_{0i} ,y_{0i} } \right)$ represents the position of the $i$-th threat center. $T\left( {x,y} \right)$ represents the height information corresponding to the threat. $T_{i}$ is the ability of the corresponding threat $i$ to act. $x_{si}$ and $y_{si}$ both represent attenuation coefficients. The superposition method of terrain data and threat altitude data is represented by Eq. (4).

4 $$ Z\left( {x,y} \right) = P\left( R \right) \cdot T\left( {x,y} \right) + H\left( {x,y} \right) $$

In Eq. (4), $Z\left( {x,y} \right)$ is the terrain data obtained by superimposing the threat and terrain height. $P\left( R \right)$ is the probability of the threat with a distance of $R$ from the $i$-th threat center. $H\left( {x,y} \right)$ is the terrain height value before data stacking. UAV needs to perform low altitude flights to reduce the probability of being detected during flight missions. For this purpose, three different threat models are constructed, namely terrain, radar threat, and electronic interference threat models. The mathematical model of terrain threat is represented by Eq. (5).

5 $$ \begin{gathered} H\left( {x,y} \right) = \sin \left( {y + a} \right) + b \cdot \sin x \hfill \\ + \cos \left( {d \cdot \sqrt {x^{2} + y^{2} } } \right) + g \cdot \cos y \hfill \\ \end{gathered} $$

In Eq. (5), $a,b,d,e,f,g$ are constants. The terrain undulations are simulated by changing these constants. In general, the first consideration for UAV is radar detection when conducting flight missions in areas with radar hazardous radiation sources. The detection range of radar radiation sources determines the degree of threat to the UAV during mission execution. The relationship between radar detection probability, radar false alarm probability, and maximum radar operating range is represented by Eq. (6).

6 $$ \left\{ {\begin{array}{*{20}c} {\frac{{R_{1} }}{{R_{2} }} = \left[ {\frac{{\ln P_{1} \left( {\ln P_{fa} - \ln P_{2} } \right)}}{{\ln P_{2} \left( {\ln P_{fa} - \ln P_{1} } \right)}}} \right]} \\ {SNR = \frac{{\ln P_{fa} }}{{\ln P_{d} }} - 1} \\ \end{array} } \right. $$

In Eq. (6), $P_{fa}$ is the radar false alarm probability. $P_{d}$ is the probability of the radar detection. $P_{1}$ and $P_{2}$ represent the detection probabilities of the radar at two different distances $R_{1}$ and $R_{2}$, respectively. From this, the radar detection rate in Eq. (7) can be calculated.

7 $$ P_{d} \left( R \right) \approx \exp \left[ { - \left( {R/R_{\max } } \right)^{4} } \right] $$

In Eq. (7), $R$ represents the radial distance of UAV distance threat. $R_{\max }$ is the maximum operating range of the radar. The elevation data model of radar threat is obtained by integrating the radar threat range and detection probability, represented by Eq. (8).

8 $$ H_{radar} \left( {x,y} \right) = K_{r} \cdot \left( {R_{\max }^{2} - \left( {x - x_{0} } \right)^{2} - \left( {y - y_{0} } \right)^{2} } \right) $$

In Eq. (8), $K_{r}$ is the performance coefficient related to radar. $\left( {x_{0} ,y_{0} } \right)$ is the coordinate of the radar center position. Electronic interference danger refers to a killing method in the enemy's prevention and control system that interferes with the recorded electromagnetic pulses of flying targets. UAV mainly uses GPS for navigation. But when the navigation signal is interfered with, UAV will lose control. The elevation data of the electronic interference threat model satisfy Eq. (9).

9 $$ \left( {x - x_{m} } \right)^{2} + \left( {y - y_{m} } \right)^{2} + \left( {H_{m} \left( {x,y} \right) - z_{m} } \right)^{2} = R_{m}^{2} $$

In Eq. (9), $\left( {x_{m} ,y_{m} ,z_{m} } \right)$ is the center point coordinate of the electronic interference source. $H_{m} \left( {x,y} \right)$ is the elevation data. After overlaying the electronic interference model with the terrain, Eq. (10) is obtained.

10 $$ H^{\prime}_{m} \left( {x,y} \right) = \sqrt {R^{2} - \left( {x - x_{m} } \right)^{2} - \left( {y - y_{m} } \right)^{2} } + z_{m} + H\left( {x,y} \right) $$

**Figure 2:**
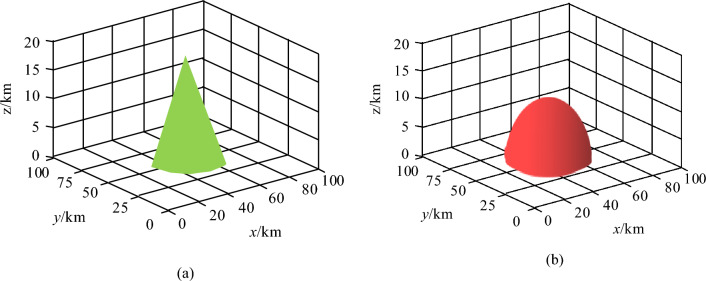
3D rendering of radar threat and electronic jamming threat. (**a**) 3D Map of Radar Threat, (**b**) 3D diagram of electronic interference threat.

After constructing three threat models, the study applies them to unmanned trajectory planning as a threat factor that needs to be avoided during UAV mission execution. The 3D effects of radar threat and electronic interference threat are shown in Fig. 2.

### Unmanned aerial vehicle trajectory planning based on i-gwo

**Figure 3:**
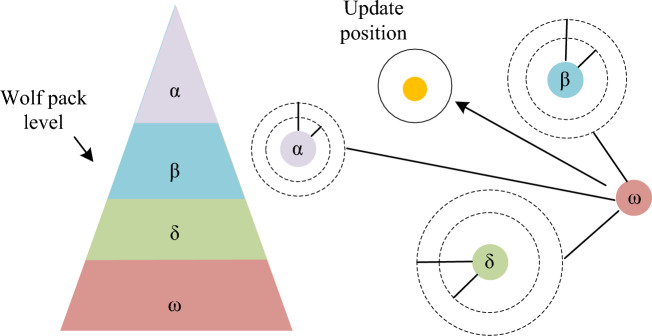
Schematic diagram of the grey wolf algorithm. (**a**) 3D map of radar threat. (**b**) 3D diagram of electronic interference threat.

The trajectory planning of UAV clusters in a 3D environment is a highly challenging and multi-constrained optimization problem. The study uses I-GWO to solve trajectory planning^19–21^. GWO has the advantages of simple structure, fewer required adjustment parameters, and good balance, so it is widely used in various fields. Wolf packs generally engage in collective hunting, and the social hierarchy of gray wolves plays an important role^22,23^. Under the leader-ship of the head wolf, stronger gray wolves assist in decision-making, while old and weak gray wolves accept the leader-ship and protection of other gray wolves. The hunting process of gray wolves is divided into three steps: chasing and tracking prey, surrounding and chasing prey, and attacking prey. The specific GWO is shown in Fig. 3.

The behavior of gray wolves chasing prey during hunting is defined as Eq. (11).

11 $$ \left\{ {\begin{array}{*{20}c} {\vec{D} = \left| {\vec{C} \cdot \vec{D}_{p\left( t \right)} - \vec{X}_{\left( t \right)} } \right|} \\ {\vec{X}_{{\left( {t + 1} \right)}} = \vec{X}_{P\left( t \right)} - \vec{A} \cdot \vec{D}} \\ \end{array} } \right. $$

In Eq. (11), $\vec{D}$ is the distance between the individual gray wolf and its prey. $\vec{C}$ and $\vec{A}$ are vector coefficients. $\vec{X}_{\left( t \right)}$ and $\vec{X}_{P\left( t \right)}$ are vectors for the position of the gray wolf and prey, respectively. $t$ is the number of iterations. $\vec{X}\left( {t + 1} \right)$ is the equation for updating the position of gray wolves. $\vec{D}_{P(t)}$ is the distance between the prey's position and the center point. These two vector coefficients are represented by Eq. (12).

12 $$ \left\{ \begin{gathered} \vec{A} = 2\vec{a} \cdot \vec{r}_{1} - \vec{a} \hfill \\ \vec{C} = 2\vec{r}_{2} \hfill \\ \end{gathered} \right. $$

In Eq. (12), $\vec{a}$ is the convergence factor, and its value decreases linearly from 2 to 0 with the number of iterations. $\vec{r}_{1}$ and $\vec{r}_{2}$ are random numbers within [0, 1]. The mathematical model of grey wolf tracking individuals is represented by Eq. (13).

13 $$ \vec{D}_{\varsigma } \left( t \right) = \left| {\vec{C} \cdot \vec{X}_{\varsigma \left( t \right)} - \vec{X}_{\left( t \right)} } \right| $$

**Figure 4:**
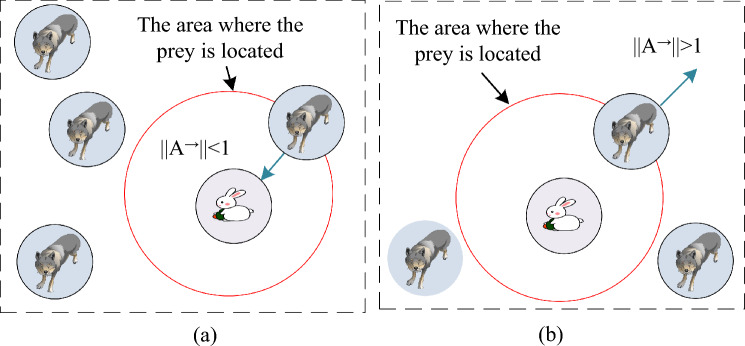
Attack and search processes of the grey wolf algorithm. (**a**) Attack, (**b**) Search.

In Eq. (13), $\vec{D}_{\varsigma }$ represents the distance between the gray wolf $\varsigma$ and other wolves. $\vec{X}_{\varsigma }$ is the current position vector of $\varsigma$. $\vec{X}_{(t)}$ is the position of a gray wolf, which will update during the iteration process with the position of the optimal wolf. The attack and search processes of GWO are shown in Fig. 4.

Although GWO has superior performance, the lack of correlation between parameter changes in each iteration of the algorithm results in low convergence and easy trapping in local optima^24–26^. Therefore, inspired by the pheromone of Ant Colony (AC) algorithm, this study introduces pheromone factors into GWO to enhance cooperation between wolves^27^. In addition, pheromones are optimized based on the hierarchical system of wolf packs and then used to guide subsequent iterative operations. The increment of pheromones is represented by Eq. (14).

14 $$ \Delta \tau_{i,i + 1} (t) = \frac{{Q_{P} }}{{d_{i,i + 1} }},P \in \left\{ {\alpha ,\beta ,\delta } \right\} $$

In Eq. (14), $Q_{P}$ represents the influence of new pheromones left by the gray wolf. $P$ is the wolf pack level. $\alpha ,\beta ,\delta$ represent the first, second, and third social levels of wolf packs. $d_{i,i + 1}$ is the length from paths $i$ to $i + 1$. $\Delta \tau_{i,i + 1} \left( t \right)$ is the pheromone increment of the path points $i$ to $i + 1$ passed by the three levels of wolves at $t$-th iteration. In each iteration, the previously existing pheromones will decay, while the pheromones of the new optimal wolf will be superimposed. Therefore, the update of pheromone concentration is represented by Eq. (15).

15 $$ \left\{ {\begin{array}{*{20}c} {\tau_{i,i + 1} \left( {t + 1} \right) = \left( {1 - \rho } \right) \cdot \tau_{i,i + 1} \left( t \right) + \Delta \tau_{i,i + 1} \left( t \right)} \\ {\rho \in \left[ {0,1} \right]} \\ \end{array} } \right. $$

In Eq. (15), $\tau_{i,i + 1} \left( t \right)$ represents the concentration of pheromones. $\rho$ is the attenuation factor of pheromones. Pheromone attenuation factors determine the rate at which pheromones decrease over time. The design of different attenuation factors will play different roles in the scene. In practical applications, the choice of attenuation factor needs to be weighed according to the characteristics of the specific problem. A smaller attenuation factor can be selected for scenarios that require fast convergence. A larger attenuation factor can be selected for scenarios that require higher diversity. In addition, it should consider using different attenuation factors at different stages of the algorithm to maintain diversity while increasing convergence speed. Fast convergence scenarios tend to occur in applications where the time requirements are strict and the problem space is relatively simple. For example, in real-time control systems or online optimization tasks, the algorithm needs to find the best solution in a short time to meet the real-time requirements of the system. Scenarios requiring high diversity usually appear in complex problem space, where there may be multiple local optimal solutions, but the global optimal solutions are difficult to find directly. For example, in areas such as pattern recognition, image processing, or machine learning, algorithms need to process large amounts of data and features, and they need to extract useful information from them for decision-making or classification. In these scenarios, maintaining a high diversity helps the algorithm to explore a broader problem space, avoid falling into local optima prematurely, and thus improve the possibility of finding a global optimal solution.

Only the optimal three wolves are allowed to leave pheromones on the path points they pass through to highlight the guiding role of the local optimal trajectory obtained in each iteration and reduce the computational complexity of the algorithm. At each path point, each wolf may have several feasible alternative path points when generating the next path point. The probability of each feasible path point being selected is represented by Eq. (16).

16 $$ P_{{i,h_{i}^{t} }} \left( t \right) = \frac{{\left[ {\tau_{{i,h_{i} }} \left( t \right)} \right]^{\gamma } \left[ {\eta_{{i,h_{i} }} \left( t \right)} \right]^{\xi } }}{{\sum\nolimits_{{h_{i} \in H\left( i \right)}} {\left[ {\tau_{{i,h_{i} }} \left( t \right)} \right]^{\gamma } \left[ {\eta_{{i,h_{i} }} \left( t \right)} \right]^{\xi } } }} $$

**Figure 5:**
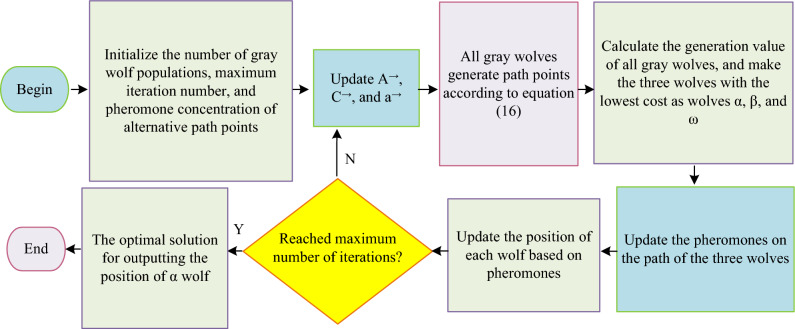
I-GWO flow after introducing pheromone factors.

In Eq. (16), $H\left( i \right)$ is the set of all feasible next path points on the $i$-th path point. $\gamma$ and $\xi$ are normal numbers, meaning the preference level of grey wolves for pursuing and selecting the current path, respectively. $\eta_{{i,h_{i} }}$ is the heuristic factor. $\tau_{{i,h_{i} }} \left( t \right)$ is the concentration of pheromones. I-GWO is shown in Fig. 5.

As mentioned earlier, the study uses the line method to select UAV trajectory points, dividing the starting point and target point into $D$ equal parts. The total cost function of UAV cluster trajectory planning is composed of fuel and threat costs. The threat cost is inversely proportional to the distance between the threat source point and UAV. In summary, the path of UAV is solved through I-GWO, and the least costly waypoint is found during the iteration.

Since the traditional GWO highly depends on the initial value of parameters compared with other algorithms, the study screened the optimal initial parameter combination through the experimental test method, and finally obtained the parameters of the I-GWO, as shown in Table 1^28^.

**Table 1:** Initial parameter settings for I-GWO.

Parameter	Value
Population size	50
Pheromone attenuation factor	0.25
$\gamma$	10^4^
$\xi$	14
$Q_{\alpha }$: $Q_{\beta }$:$Q_{\delta }$	1.0:0.5:0.3
Weight coefficients $\lambda_{1}$, $\lambda_{2}$, and $\lambda_{3}$ of the cost function	10^–2^, 10^–1^, and 10^–4^

### Optimization strategy for trajectory planning model based on reinforcement learning

As the UAV navigation environment becomes increasingly complex, sudden threats will also increase, which will lead to the failure of swarm intelligence algorithms for trajectory replanning^29,30^. Therefore, the study utilizes reinforcement learning for trajectory replanning and proposes an improved strategy for actual trajectory planning using Deep Deterministic Policy Gradient Algorithm (DDPG). This can achieve dynamic optimization of trajectory planning models. When UAV is conducting trajectory replanning, it can obtain information on position, relative position, speed, and other aspects through sensors and intelligence. Therefore, combining these three aspects of information can represent the state information at any time during UAV trajectory replanning, represented by Eq. (17).

17 $$ S_{t} = \left( {\left[ {x_{u,t} ,y_{u,t} ,h_{u,t} } \right],\left[ {dx_{t} ,dy_{t} ,dh_{t} } \right],\left[ {v_{x,t} ,v_{y,t} ,v_{z,t} } \right]} \right) $$

In Eq. (17), $\left[ {x_{u,t} ,y_{u,t} ,h_{u,t} } \right]$ represents the coordinate position of UAV in flight space at time $t$. $\left[ {dx_{t} ,dy_{t} ,dh_{t} } \right]$ is the relative position between UAV and the target. $\left[ {v_{x,t} v_{y,t} v_{z,t} } \right]$ represents the sub-velocity of UAV in three directions. The action of UAV is represented by Eq. (18).

18 $$ A_{t} = \left( {\varphi_{t} \theta_{t} } \right) $$

In Eq. (18), $\varphi_{t}$ is the direction angle of UAV. $\theta_{t}$ is the pitch angle of UAV. The UAV motion used in the study adopts a constant acceleration model for motion. To improve the convergence of trajectory replanning, non-sparse rewards are set up. When facing dynamic threats in UAV, it is necessary to reach the target location as quickly as possible while avoiding threats such as radar detection and electronic interference. The selected threat models are all related to the distance of UAV. Therefore, the study considers real-time distance as a reward factor for UAV trajectory replanning. After selecting the distance factor as a reward, the study subdivides it into four types: negative rewards for voyage, boundary, and threat, and positive reward for arrival. The negative reward for the voyage is represented by Eq. (19).

19 $$ R_{hancheng} = \left\{ {\begin{array}{*{20}c} { - \left\| {\hat{N}\left( d \right)} \right\|,d \ge l_{\max } } \\ { - 2,d < l_{\max } } \\ \end{array} } \right. $$

In Eq. (19), $\hat{N}\left( \cdot \right)$ is the normalization formula. $d$ is the flight range that has been flown by UAV. $l_{\max }$ is the maximum flight range corresponding to UAV carrying fuel. When reaching the target point, the system will provide feedback on a positive reward, represented by Eq. (20).

20 $$ R_{daoda} = \left\{ {\begin{array}{*{20}c} {2 - \left\| {\hat{N}\left( {P_{t,t} - P_{u,t} } \right)} \right\|,if\left\| {P_{t,t} - P_{u,t} } \right\| \le \phi_{\max } } \\ { - \left\| {\hat{N}\left( {P_{t,t} - P_{u,t} } \right)} \right\|,if\left\| {P_{t,t} - P_{u,t} } \right\| > \phi_{\max } } \\ \end{array} } \right. $$

**Figure 6:**
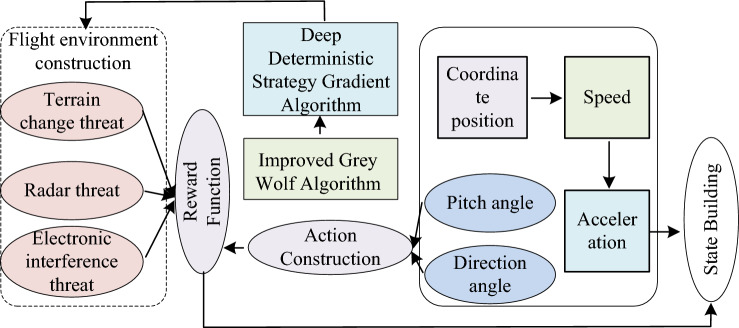
Improved trajectory planning model.

In Eq. (20), $P_{u,t}$ represents the location where task UAV is executed. $P_{t,t}$ is the location of the target point. $\phi_{\max }$ is the maximum detection distance of UAV. Similarly, boundary and threat negative rewards are set, and the final reward is obtained by multiplying the weights of each reward and adding them together. The improved trajectory planning model is shown in Fig. 6.

The study combines value functions with policy gradients and adopts DDPG for model dynamic optimization to better face dynamic and sudden threats. This algorithm is divided into two parts: policy network and value function network, which evaluate the actions generated by the policy network using the value function network. The action selection is optimized by generating error correction, represented by Eq. (21).

21 $$ \mathop {\lim }\limits_{\sigma \downarrow 0} \nabla J\left( {\pi_{{\mu_{\theta } ,\sigma }} } \right) = \nabla_{\theta } J\left( {\mu_{\theta } } \right) $$

In Eq. (21), $\theta$ is the direction angle. $\nabla J_{\theta } \left( {\mu_{\theta } } \right)$ is the gradient of the policy parameter corresponding to the objective function. $\sigma$ is a gradient. $\mu_{\theta }$ is a deterministic strategy. $\pi_{{\mu_{\theta } ,\sigma }}$ is a random strategy. The updates of the value function and policy networks are represented by Eq. (22).

22 $$ \left\{ \begin{gathered} \nabla_{{\theta^{\mu } }} J\left( {\theta^{\mu } } \right) = \frac{1}{N}\sum\limits_{i} {\nabla_{a} Q\left( {s,a|\theta^{Q} } \right)\nabla_{{\theta^{\mu } }} \mu \left( {s|\theta^{\mu } } \right)} \hfill \\ s = s_{i} ,a = \mu \left( {s_{i} } \right) \hfill \\ L^{\prime} = \frac{1}{N}\sum\limits_{i} {\left( {y_{i} - Q(s,a|\theta^{Q} )} \right)^{2} } \hfill \\ \end{gathered} \right. $$

**Figure 7:**
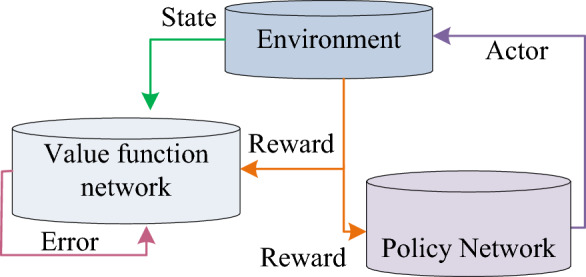
The algorithmic framework for combining value function networks and policy networks.

In Eq. (22), $\mu \left( {s|\theta^{\mu } } \right)$ and $Q\left( {s,a|\theta^{Q} } \right)$ represent the value function and policy networks, respectively. $\theta^{\mu }$ and $\theta^{Q}$ are parameters of two networks, respectively. $s$ is the UAV status information. $N$ is a real number based on the environment. $L^{\prime}$ is the updated value of the policy network. The algorithm framework is shown in Fig. 7.

**Figure 8:**
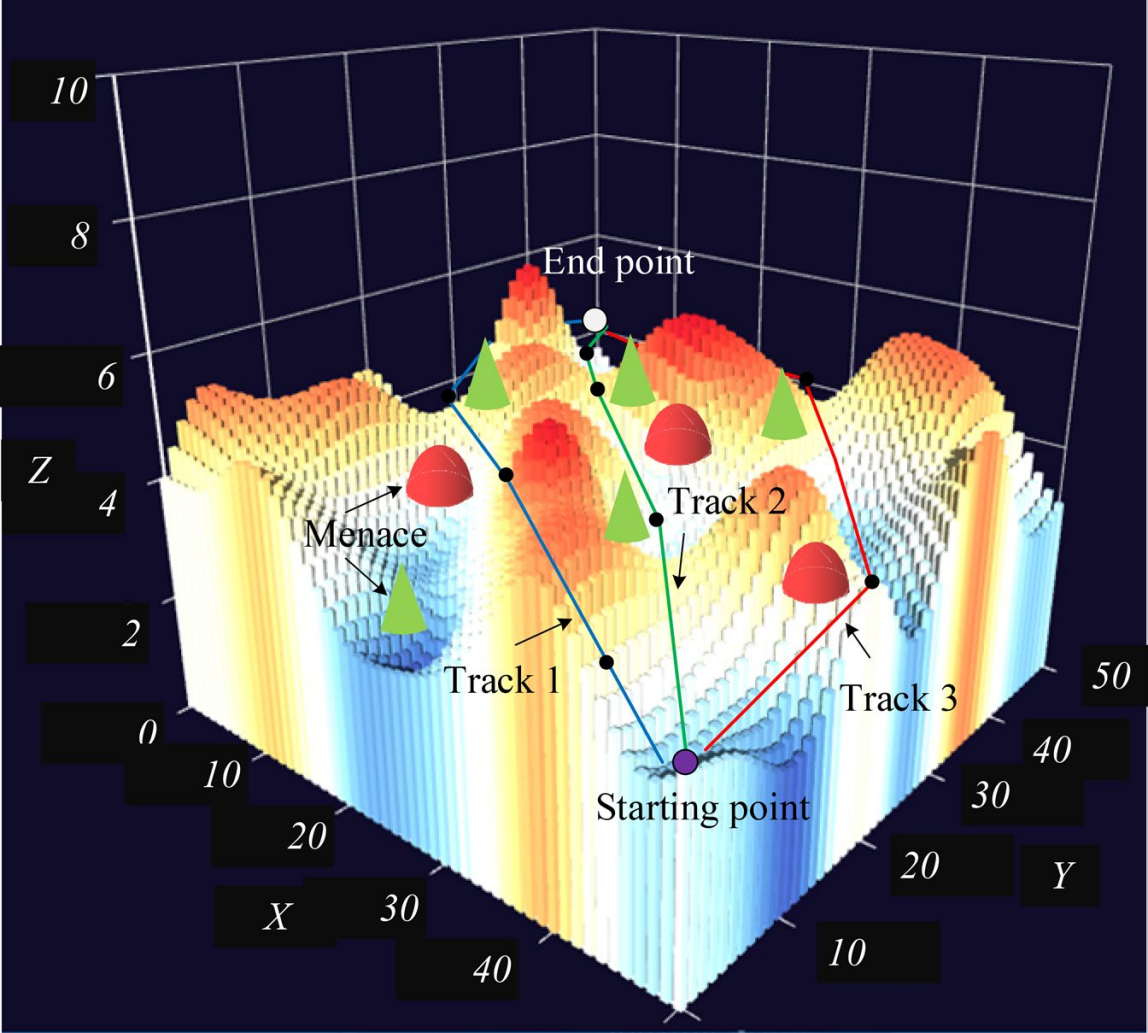
Three-dimensional representation of system operation.

The hyper-parameter adjustment in this algorithm will directly affect the convergence effect of the final model, and there is a high sensitivity issue. Therefore, the study combines it with neural networks to optimize their grid parameters and ultimately achieve an improvement in training accuracy. The reward factors obtained are used as the fitness function, and the weights are optimized using neural networks to improve the fitting effect. With more UAV, the system actions will require more complex and refined planning and coordination. When assigning tasks to each UAV, Particle Swarm Optimization (PSO) algorithm is used to find the optimal task assignment scheme to improve the efficiency and performance of the system. The multi-agent cooperative optimization technology is studied to further improve the precision and cooperative efficiency of UAV flight path planning. Specifically, the technology realizes the overall optimization of the UAV swarm by building a multi-agent system. Meanwhile, each UAV is treated as an independent agent, and the information interaction and collaborative decision-making between the agents is utilized. Figure 8 presents the operation of the system in a 3D form.

### Performance analysis of unmanned aerial vehicle swarm trajectory planning optimization model based on i-gwo

**Figure 9:**
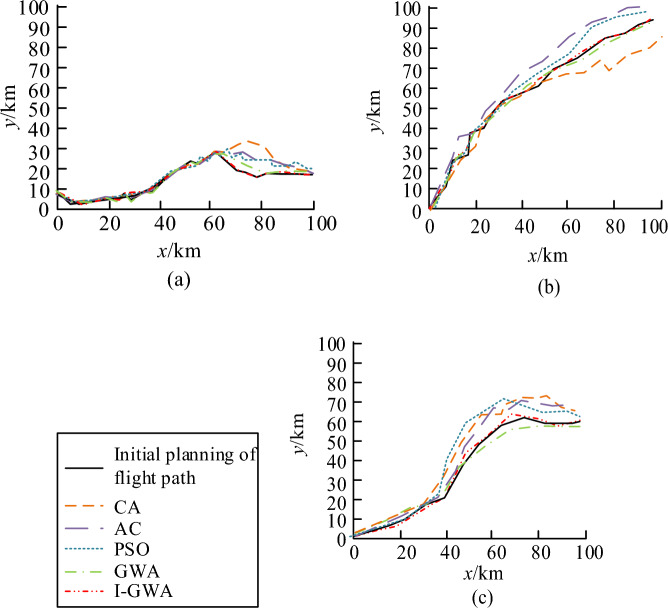
Replanning results of species algorithm. (**a**) Yamagata 1. (**b**) Yamagata 2. (**c**) Yamagata 3.

A series of simulation experiments were designed in MATLAB to verify the performance of the proposed trajectory planning model. A comparative experiment was conducted with the currently popular centralized optimization algorithms to verify the improvement effect of GWO and the rationality of using this algorithm for trajectory planning. The scale of the environment has a significant influence on the result of UAV cluster track planning. The larger scale means that the UAV has more space for flight path planning, but it also increases the complexity and difficulty of flight path planning. In a vast environment, UAV needs to take into account more terrain, obstacles, and potential threats. The scale of the environment will also affect the detection and communication range of the UAV. In larger environments, UAV may need to fly longer distances to reach their target points, while maintaining communication with other UAV or command centers becomes more difficult. In three different mountain conditions, various algorithms were used for replanning the trajectory, including the Cuckoo Algorithm (CA), AC algorithm, PSO, GWO, and I-GWO. The comparison results are shown in Fig. 9.

**Figure 10:**
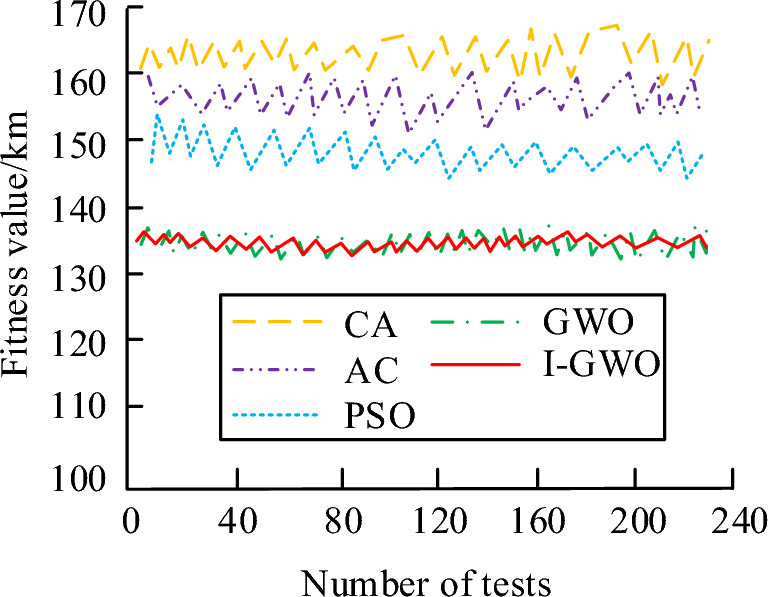
Stability analysis of several algorithms.

In Fig. 9, I-GWO and GWO corresponded to a re-planned trajectory length of 62.54 km and 60.81 km, respectively, with a difference of 1.73 km, indicating an increase in the range of the algorithm after improvement. Compared to these two algorithms, the other three algorithms had a re-planned trajectory length greater than 80 km, which clearly required an additional range of 19.19 km. The range of the CA reached 105.22 km, which did not meet the requirement of the shortest track. The stability simulation analysis was conducted on five algorithms to further explore the performance of I-GWO in Fig. 10.

According to Fig. 10, the maximum difference between fitness values in I-GWO was 5.45, while the traditional GWO was 5.88, with a difference of 0.43 between them. Compared with the other three optimization algorithms, I-GWO had a lower optimal fitness value and a smaller fluctuation range. Therefore, the stability of I-GWO was not significantly different from before improvement, but it had higher stability compared to other algorithms. An increase in the number of threats will make it more difficult to plan UAV flight paths. When there are more threats in the environment, UAV needs to plan their flight paths more finely to avoid those threat areas. The increased threats have an impact on UAV flight performance. The increased threat has a detrimental effect on the flight performance of UAVs, and in order to avoid threat areas, UAVs may need to maneuver and adjust altitude frequently, which increases their power consumption and flight time. In cluster operations, UAV needs to cooperate and cooperate with other UAV to jointly complete combat tasks together. However, when there are a large number of threats in the environment, coordination and cooperation between UAV may become more complex and difficult, and may even lead to communication interruptions and coordination failures. A study was conducted to compare the trajectory planning effects of each algorithm in three different scenarios to test the trajectory planning effectiveness of each optimization algorithm. There were 5 threat sources in scenario 1, 7 in scenario 2, and 10 in scenario 3. The comparison results are shown in Table 2.

**Table 2:** Track planning results of each algorithm in three scenarios.

Algorithm		Average cost	Least cost	Maximum cost	Cost standard deviation	Average time (s)	Track length (km)
Scenario 1	CA	293.45	289.34	294.89	1.15	12.34	81.44
	AC	287.56	282.45	290.12	1.54	10.92	78.56
	PSO	288.95	283.71	291.44	1.42	10.17	77.78
	GWO	280.45	274.67	282.36	1.23	9.63	72.42
	I-GWO	281.58	276.83	283.15	0.94	5.45	70.18
Scenario 2	CA	291.56	289.45	294.56	1.23	12.88	81.67
	AC	287.45	282.08	290.47	1.57	11.21	78.76
	PSO	288.13	284.02	291.63	1.44	10.73	78.00
	GWO	280.73	274.98	282.72	1.26	10.04	72.54
	I-GWO	281.25	277.03	283.43	0.98	5.78	70.32
Scenario 3	CA	293.44	289.95	294.87	1.30	13.00	81.88
	AC	287.92	282.28	290.74	1.61	11.42	78.90
	PSO	288.26	283.92	292.00	1.48	10.96	78.21
	GWO	280.43	275.10	282.88	1.29	10.21	72.73
	I-GWO	281.62	277.24	283.65	1.00	5.92	70.51

**Figure 11:**
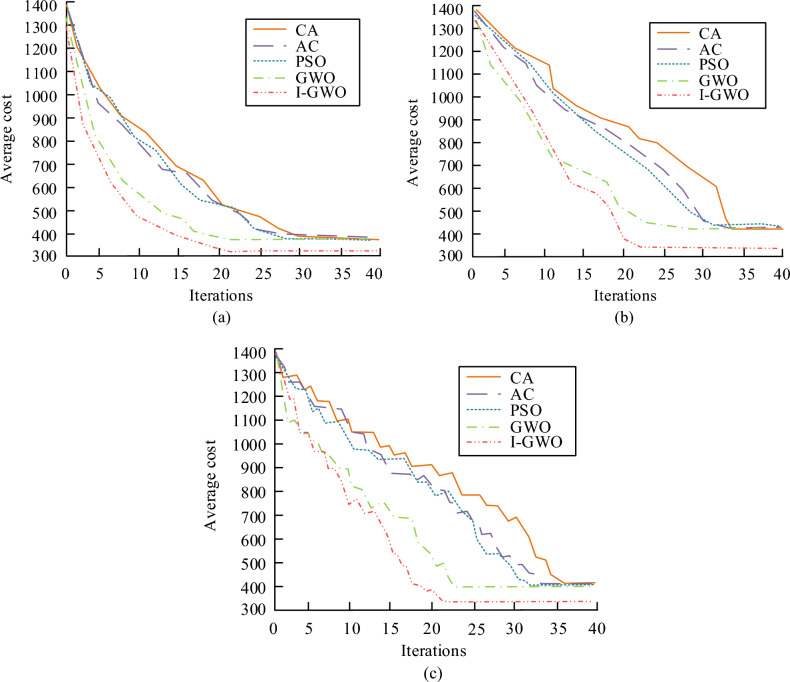
Comparison of convergence performance of several optimization algorithms. (**a**) Scenario 1. (**b**) Scenario 2. (**c**) Scenario 3.

As the number of threat sources increased, the average cost and trajectory length of each algorithm increased. In the simpler scenario 1, the average generation value and cost standard deviation obtained by I-GWO and the original GWO were close to those obtained by AC. In more complex scenarios 2 and 3, I-GWO had significantly better values than the other algorithms in all indicators except for the running time. As the complexity of the scene increased, I-GWO had a more significant advantage in trajectory planning. To further test the superiority of I-GWO, the study conducted training iterations on each algorithm in three scenarios and records the training results in Fig. 11.

**Figure 12:**
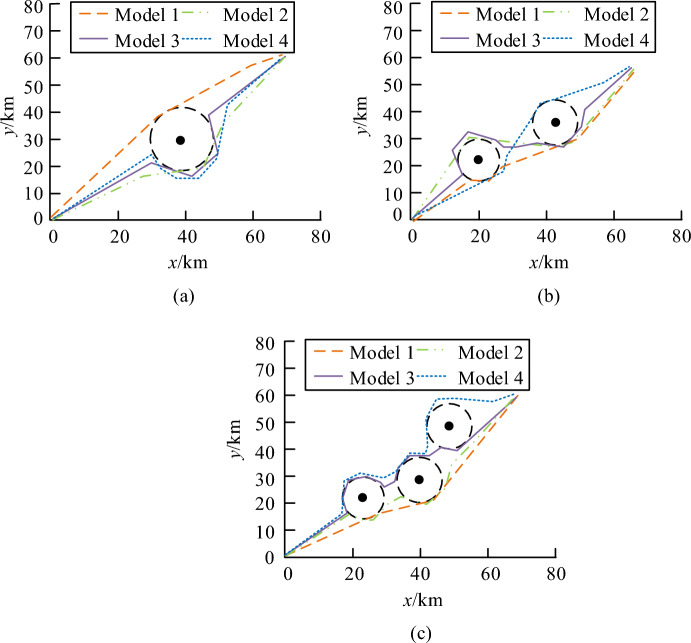
Track planning results of four models in different sudden threat regions, (**a**) Single threat zone, (**b**) Dual threat zone, (**c**) Multiple threat zones.

From Fig. 11, I-GWO achieved the target cost and began to converge after only about 20 iterations. Although the convergence speed of traditional GWO was basically the same as that of the improved algorithm, the convergence accuracy was lower compared to the improved algorithm. In addition, the other three algorithms all converged after about 30 iterations, and the convergence accuracy was basically the same as traditional GWO. Therefore, I-GWO had the high convergence accuracy. In response to sudden dynamic threats, research improved the model using reinforcement learning methods. This study compared the UAV swarm trajectory planning model designed by this research (Model 1) with existing advanced dynamic threat UAV trajectory planning models to test the improvement effect of the model and the collaborative planning effect of the improved model in solving tasks. The comparative models included a UAV online trajectory planning model based on a real-time search prediction algorithm (Model 2), a dynamic trajectory planning model based on an improved constrained differential evolution algorithm (Model 3), and a trajectory planning model based on an improved heuristic search algorithm (Model 4). The trajectory planning results of the four models under single, double, and multiple threat zones are shown in Fig. 12.

**Figure 13:**
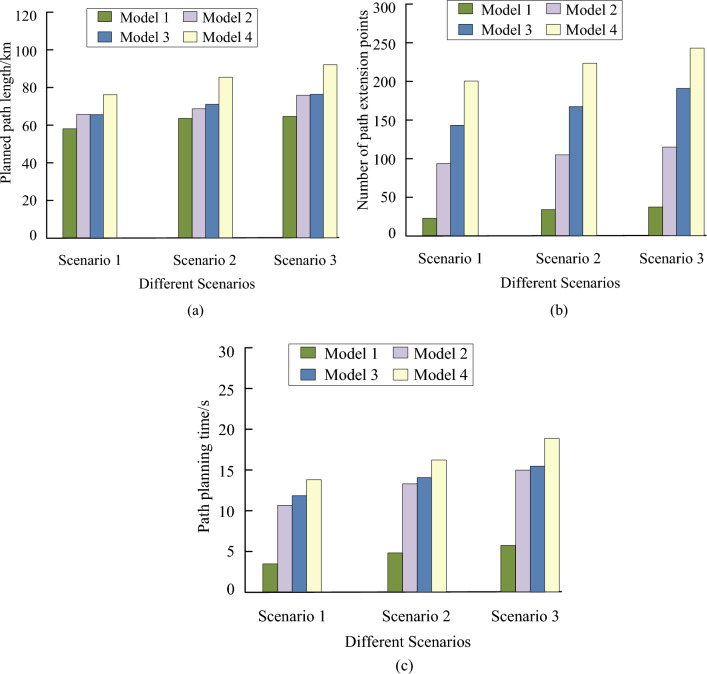
Comparison of path planning effects of the model. (**a**) Path planning length, (**b**) Path extension point, (**c**) Path planning time.

In Fig. 12a, under the same conditions, the path lengths calculated by Models 2, 3, and 4 were 75.512 km, 78.348 km, and 80.457 km, respectively. The path length of Model 1 was 58.476 km, which was significantly smaller than other models. In Fig. 12b, when encountering dual burst threats, the planned paths of Models 2, 3, and 4 were 76.412 km, 79.445 km, and 81.025 km, respectively, while the planned path length of Model 1 was 60.512 km. In Fig. 12c, the planned paths for models 2, 3, and 4 were 81.545 km, 86.941 km, and 90.441 km, respectively. The path curve calculated by Model 1 met the smoothness requirements of UAV navigation paths, and the planned path length was significantly smaller than other models. This study compared the path planning length, number of extension points, planning time, and heading changes of the four models in three scenarios to further compare and analyze the performance of the models. The specific results are shown in Fig. 13.

**Figure 14:**
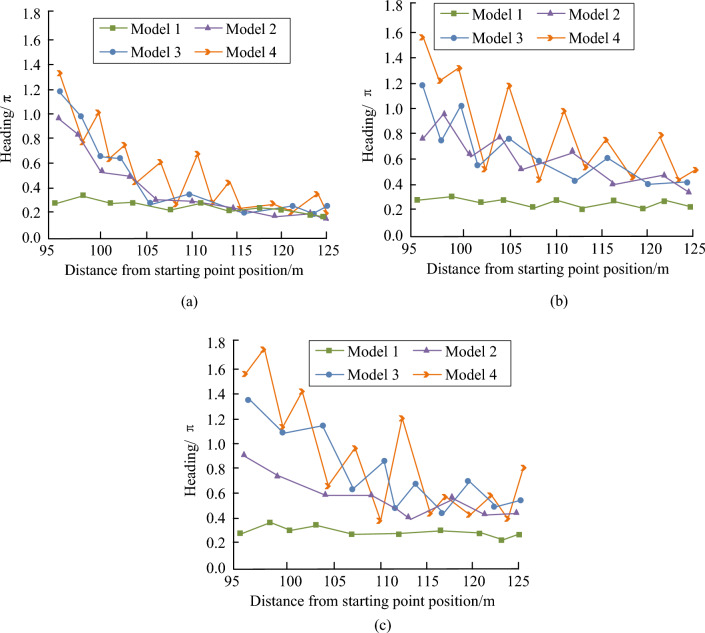
Comparison of heading changes among different models. (**a**) Scenario 1. (**b**) Scenario 2. (**c**) Scenario 3.

According to Fig. 13, in terms of path planning length, Model 1 calculated the shortest path length. In three scenarios, the average planning length of Model 1 was reduced by 18% compared to Model 2. Compared to Models 3 and 4, the planning length of Model 1 had decreased by 20% and 23%, respectively. In terms of planning time and the number of path extension points, the indicator values of Model 1 were smaller than those of other models. This indicated that Model 1 had better real-time performance compared to other models, and fewer path extension points could ensure better performance of UAV in adjusting flight attitude and speed. In addition, the experiment tested whether these models had extreme situations when sudden threats appeared to test the path heading changes of these four models in three scenarios. The changes in heading are shown in Fig. 14.

**Figure 15:**
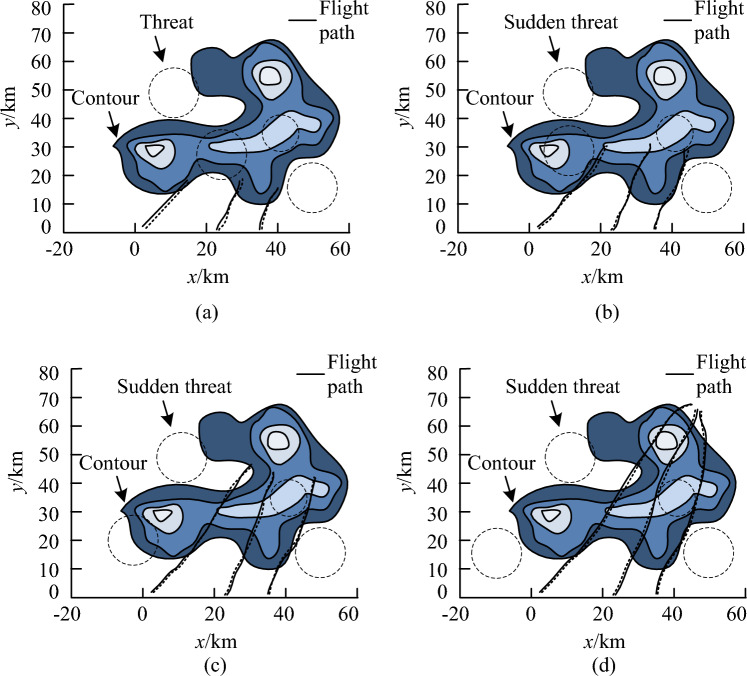
Dynamic trajectory planning results of the model. (**a**) 200th second. (**b**) 400th second. (**c**) 600th second. (**d**) 800th second.

According to Fig. 14, the curve of Model 1 changed smoothly and the heading fluctuated between 0.2 and 0.4π. However, Models 2, 3, and 4 all exhibited significant heading changes. Compared to Model 1, the heading change was relatively small and the flight process was relatively stable. This proved that Model 1 could ensure the normal flight of UAV in a real flight environment. After the above comparison, Model 1 has excellent planning performance. Research was conducted on using Model 1 for dynamic planning in simulation software to further visualize and verify the planning effectiveness of Model 1. The planning results are shown in Fig. 15.

According to Fig. 15, Model 1 effectively avoided known static threats and sudden mobile threats while tracking the reference trajectory, and the trajectory was smooth. Therefore, Model 1 had good trajectory planning performance and could help UAV change its trajectory in real-time.

In order to test the performance of the research design method in the scenario of large unmanned aerial vehicles (UAVs), and to test the scalability of the research method, an experiment was carried out. The experiment starts with a large drone swarm scenario, consisting of dozens of drones that need to work together to complete a task in a complex dynamic environment. These drones not only need to avoid static obstacles, but also need to deal with sudden mobile threats. The experiment applied Model 1 to this scenario to verify its planning performance in a large-scale drone swarm. The experimental results are shown in Table 3.

**Table 3:** Experimental Results of Model 1 in Large-scale UAV Swarm Scenarios.

Serial number	Number of UAVs	Average planned length (km)	Average planning time (s)	Average number of path extension points	Average course change (rad)	Success rate (%)
1	20	62.34	5.42	87	0.31	99.74
2	40	65.12	7.15	105	0.32	98.56
3	60	68.56	9.28	128	0.32	97.48
4	80	71.34	11.45	152	0.34	97.02
5	100	74.12	13.67	178	0.35	96.58

From Table 3, with the increase of the number of UAVs, the average planning length, average planning time and average number of path extension points all increase, but the average course change remains relatively stable. This shows that model 1 can still maintain a stable course change in the scenario of large UAVs, so as to ensure the flight stability of UAVs. At the same time, the success rate decreased slightly when the number of drones increased, but the overall level remained high, indicating that model 1 still has a high planning success rate in large-scale scenarios. Based on the above experimental results, it can be concluded that model 1 of the research design performs well in the scenario of large UAVs, and can effectively cope with complex dynamic environment and realize collaborative task planning. At the same time, model 1 has good scalability and can adapt to different scale UAV swarm scenarios. This provides strong support for the future application of large-scale UAVs.

## Conclusion

At present, trajectory planning techniques have been widely applied, but the current trajectory planning technology cannot achieve ideal planning results. To this end, the study utilized the pheromone factor I-GWO and combined it with DRL to achieve trajectory optimization of UAV dynamic and static states. Through the experimental analysis, I-GWO and GWO had a re-planned trajectory length of 62.54 km and 60.81 km, respectively, with a difference of 1.73 km. After the improvement, the algorithm's range was increased. Compared to other algorithms, the planned path length of I-GWO was significantly smaller. I-GWO achieved the target cost in about 20 iterations and began to converge. Although the convergence speed of traditional GWO was basically the same as that of the improved algorithm, the convergence accuracy was lower compared to I-GWO. The path length of Model 1 was 58.476 km, which was significantly smaller than other models. As the number of experiments in Model 1 increased, the curve changed smoothly and the heading fluctuates between 0.2 and 0.4π. There was no obvious sudden change in heading. Model 1 could effectively avoid obstacles while tracking the reference trajectory, while achieving low-cost trajectory planning, and the trajectory was relatively smooth. Therefore, the constructed model has excellent performance in UAV trajectory planning and can complete effective trajectory replanning flight tasks in complex environments. This study only conducted small-scale trajectory planning during the UAV flight. However, in practical scenarios, the scale of UAV swarm will be relatively large. Therefore, further exploration of the operational performance of the model on a larger scale is needed in future research. At present, research only simplifies UAV into particle models to control their pitch and yaw angles. However, there is no more detailed consideration of the specific models of UAV. In future research, more specific physical models can be added to further optimize their trajectory problems. In practical applications, unmanned opportunities are subject to many uncertain factors, such as tornado, rainstorm, and other extreme weather that affect the driving of UAV, sudden changes in tasks, insufficient battery power of UAV and other uncertain factors. In future research, these factors can be further incorporated into the model to improve the robustness of the planning model.

Windy not only affects the flight speed and stability of UAV, but also poses additional challenges to their flight path planning. The study needs to incorporate the wind effect into the consideration of flight path planning to solve this problem. First, the UAV dynamics model needs to be further refined. The wind field model is introduced to simulate the wind effect. Future work can simulate different wind field conditions, including wind speed, wind direction, and spatial distribution of wind field to compare the processing time of different algorithms under windy conditions. The time required for each algorithm can be recorded to complete the track planning. The efficiency of different algorithms when dealing with windy conditions can be evaluated by comparing these time data.

## Data Availability

The datasets used and/or analyzed during the current study available from the corresponding author on reasonable request.
